# Assessment of eligibility for thrombolysis in acute ischaemic stroke patients in Morocco

**DOI:** 10.11604/pamj.2020.36.351.22599

**Published:** 2020-08-26

**Authors:** Mohamed Acherqui, Hajar Khattab, Younes Habtany, Rim Amzil, Salma Bellakhdar, Hicham El Otmani, Bouchra El Moutawakil, Mohammed Abdoh Rafai

**Affiliations:** 1 Neurological Department, IBN ROCHD University Hospital, Casablanca, Morocco

**Keywords:** Eligibility

## Abstract

**Introduction:**

intravenous thrombolysis with recombinant tissue plasminogen activator (rTPA) is an approved treatment for acute ischaemic stroke (AIS). However, its use remains low. We aimed to assess the eligibility of thrombolysis for our patients with AIS before implementing this treatment method in our teaching hospital.

**Methods:**

we conducted a prospective cross-sectional study in the emergency department of Casablanca University Hospital. We included every patient admitted for a stroke-related symptom. Delays between symptom-onset and admission and delays regarding the in-hospital evaluation of patients were recorded. Patients eligible for intravenous thrombolytic therapy were identified according to American Heart Association guidelines.

**Results:**

in all, 463 patients were included. Only 8.42% of patients were eligible for thrombolysis; 74% of patients were ineligible because of an onset-to-thrombolysis delay longer than 4.5 hours. Mean onset-to-thrombolysis time was 27.2 hours. Patients were admitted with a mean delay of 24.9 hours. The in-hospital evaluation, from admission to computerized tomography (CT) interpretation, averaged 2.3 hours in length.

**Conclusion:**

the percentage of patients eligible for thrombolysis remains very low in our structure. The majority would not have benefitted from the therapy because of an extra hospital delay far exceeding the recommended therapeutic window. To shorten our delays and increase the number of patients benefiting from thrombolysis, we must implement strategies aiming to improve the recognition, evaluation and management of patients from the general public to the neurovascular unit.

## Introduction

Stroke is the leading cause of disability, the second leading cause of death and the third leading cause of dementia worldwide [1,2]. More than 80% of stroke cases in the world are located in low- and middle-income countries, where 30% of adults are at greater risk of dying from non-communicable diseases than their counterparts in developed countries [3]. In African countries, stroke has become a major public health concern in the last decade, considering its social, economic and psychological costs [4,5]. In Morocco, its incidence has continued to increase over the years, and the ischaemic subtype makes up 70.9% of all stroke cases [6]. Intravenous thrombolytic therapy with recombinant tissue plasminogen activator (rTPA) has emerged as an approved method to treat acute ischaemic stroke (AIS) [7-9], with its efficacy and safety proven by many studies [10,11]. However, due to its several exclusion criteria, its use remains low [12,13]. In Morocco, thrombolysis rates range from 1.8% to 2.9%, and the therapy remains unavailable in many facilities [14,15]. In Casablanca, the largest city in Morocco with a population of 3.36 million, the therapy was not available prior to our study in any public hospitals. As we were about to implement it in Casablanca University Hospital, we aimed prior to doing so to assess eligibility for thrombolysis among our patients with AIS in order to identify the main causes of non-eligibility and address them simultaneously to the introduction of the therapy. In this regard, considering that time is the key factor in the management of stroke in its acute phase, our objective was also to measure pre-hospital and in-hospital delays in recognising, transporting and evaluating each AIS case.

## Methods

From January to August 2017, we conducted a prospective cross-sectional study in the neurological emergency department of Casablanca University Hospital. With more than 100 000 admissions per year, our facility is a tertiary teaching hospital with the infrastructure required to implement thrombolytic therapy. We included every patient admitted for a stroke-related symptom: sudden onset of a facial droop, motor or sensory deficit, altered speech, blurred vision, balance trouble or any related persistent neurological deficit. Patients were also included regardless of their city of provenance or delay after symptom-onset. Patients aged less than 18 years, with another diagnosis at the end of the evaluation or diagnosed with a transient ischaemic attack (TIA) were excluded from our study. A TIA was identified according to the American Heart Association/ American Stroke Association (AHA/ASA) definition as a transient episode of neurological dysfunction caused by focal brain, spinal cord or retinal ischaemia, without acute infarction. Data regarding the demographic profile, cardiovascular risk factors and history of stroke of our patients were recorded. Delays between symptom-onset and hospital arrival, between admission and neurological evaluation and between clinical evaluation and CT evaluation were also recorded. CT evaluation was defined as the time the CT was reported by the radiologist. When the time of symptom-onset was not precisely known, it was defined as the last time the patient had been observed awake and symptom-free. We chose the delay between symptom-onset and CT evaluation as equivalent to the onset-to-thrombolysis delay, considering that the therapy, if made available, would be initiated immediately after evaluation of the brain imagery.

Information about the means of transport and previous admission to other healthcare facilities was also obtained. Data regarding the clinical evaluation of the patient included vital signs and neurological findings using the National Institute of Health Stroke Scale (NIHSS). CT findings were evaluated using the Alberta Stroke Program Early CT score (ASPECT). Patients were identified as eligible for thrombolytic therapy by using American Heart Association´s (AHA) Guidelines (Table 1). Since we applied the 2013 AHA Guidelines to our study, we considered that the required onset-to-thrombolysis delay was 4.5 hours for patients younger than 80 years and 3 hours for those older than 80 years. The study did not consider the ability of our patients to afford the therapy, and no informed consent was obtained since none were to receive it. However, all the patients and/or their surrogates were informed about the data collection and the involvement in our study. Ethical approval was obtained from Casablanca University Hospital with the acceptance of the study as a doctorate thesis. All data were analysed using Epi Info 7. Quantitative data were analysed using means and standard deviations (SDs), while qualitative data were expressed in percentages with evaluation of the 95% confidence interval (CI).

**Table 1:** inclusion and exclusion criteria for thrombolysis with rTPA according to AHA 2013 Guidelines

Inclusion criteria	Exclusion criteria
Diagnosis of ischaemic stroke causing measurable neurological deficit	Significant head trauma or prior stroke in previous 3 months
Onset of symptoms < 3 hours before beginning treatment	Symptoms suggest subarachnoid haemorrhage
Onset of symptoms within 3 to 4.5 hours among patients aged <80 years	Arterial puncture at non-compressible site in previous 7 days
Age ≥ 18 years	History of previous intracranial haemorrhage
	Intracranial neoplasm, arteriovenous malformation or aneurysm Recent intracranial or intraspinal surgery
	Elevated blood pressure (systolic >85 mmHg or diastolic >110 mmHg)
	Active internal bleeding Acute bleeding diathesis, including but not limited to:
	Platelet counts <100 000/mm^3^
	Heparin received within 48 hours, resulting in abnormally elevated apt
	Current use of anticoagulants with INR >1.7 or PT >15 seconds
	Blood glucose concentration <50 mg/dL (2.7 mmol/L)
	CT demonstrates multilobar infarction (hypodensity >1/3 cerebral hemisphere

## Results

Over the eight-month observation period, we initially included 463 patients, 31 of whom were diagnosed at the end of their evaluation with haemorrhagic stroke and hence excluded from our study. The remaining 432, diagnosed as having AIS, were included. The mean age of our patients was 67 years (range: 18-100 years). Among them, 15 (3%) were aged younger than 49 years, and 97 (18%) were aged older than 80 years. Women made up 50.97% (CI: 46.32-55.61) of our patients and men 49.03% (CI: 44.39-53.68), for a sex ratio of 1.04. In all, 76.57% of the patients lived in Casablanca (CI: 72.38-80.31), while 10.6% arrived from cities located more than 100 kilometres away from Casablanca (CI: 3.07-18.34) and 1.95% from cities 300 kilometres away from our hospital (CI: 0.23-10.28). The baseline characteristics of our patients, including stroke risk factors, city of origin and type of stroke according to the Trial of Org 10172 in Acute Stroke Treatment (TOAST) are summed up in Table 2. A total of 16.2% of the patients had been previously diagnosed as having heart disease (CI: 13.02-19.95), with 5.62% known to have an atrial fibrillation (CI: 3.77-8.23), and 16% had a previous history of stroke (CI: 12.8-19.71).

**Table 2:** baseline characteristics of our patients

Patient characteristics	Percentage (95% CI)
Stroke risk factors	
Hypertension	58.96 (54.32-63.46)
Diabetes	33.26 (29.02-37.79)
Smoking	24.41 (20.61-28.63)
Dyslipidemia	17.71 (14.40-21.56)
**Main cities of origin/Distance from our facility**	
Casablanca	76.57 (72.38-80.31)
Mohammedia/30 km	4.56 (2.91-6.99)
Berrechid/45 km	3.04 (1.74-5.17)
Settat/87 km	1.95 (0.96-3.81)
Khouribga/129 km	1.30 (0.53-2.96)
**Type of stroke according to the TOAST classification**	
Cardioembolism	26.98 (18.61-36.80)
Small-vessel occlusion	18.15 (11.03-26.95)
Large-artery atherosclerosis	11.08 (5.62-18.83)
Stroke of other determined aetiology	3.01 (0.62-8.52)
Stroke of undetermined aetiology	40.78 (31.26-51.29)
**Main exclusion criteria for thrombolytic therapy**	
Onset-to-thrombolysis >4.5 hours	80.77 (69.49-86.67)
Onset-to-thrombolysis >3 hours among patients aged >80 years	14.42 (11.29-18.22)
Current use of anticoagulants	6.9 (4.72-9.81)
CT suggesting multilobar infarction	3.31 (1.89-5.62)
Elevated blood pressure (systolic >185mmHg or diastolic >110 mmHg)	2.16 (1.12-4.86)
Intracranial neoplasm, arteriovenous malformation or aneurysm	0.47 (0.08-1.89)
Significant head trauma or prior stroke in previous 3 months	0.24 (0.01-1.52)

Only 8.42% of our patients were eligible for thrombolytic therapy (CI: 6.13-11.43). The main exclusion criterium was a symptom-onset to thrombolysis delay exceeding the therapeutic window of the rTPA, representing 80.77% of all cases (CI: 69.49-86.67). Among the patients aged older than 80 years, 62.88% were non-eligible for the therapy because of a delay exceeding 3 hours. These patients comprised 14.42% of the cases (CI: 11.29-18.22). The main exclusion criteria are included in Table 2. The mean onset-to-CT-evaluation time was 27.2 hours (range: 0.7-285 h). The mean onset-to-admission delay was 24.9 hours (range: 0.5-280 h). Only 14.69% of our patients were previously evaluated and transported by emergency medical services (EMS) in the pre-hospital phase (CI: 11.64-18.36). The mean delay among those who were transported by the EMS was 15.5 hours, whereas the delay for those who chose another means of transport was 26.5 hours. 60.95% of the patients (CI: 56.32-65.41) were previously evaluated in a healthcare facility without the required infrastructure for thrombolytic therapy before being transferred to our centre. The mean onset-to-thrombolysis delay for this subgroup was 26.1 hours, versus 24.1 hours for those who were first managed in our facility. No significant difference in delays was observed between patients coming from Casablanca and those coming from cities located 100 km away from our centre. The mean door-to-thrombolysis delay was 2.3 hours (range: 0.2-19 h). The mean delay between patient admission and clinical evaluation was 14.8 minutes (range: 1-120 min), while the delay between clinical evaluation and CT evaluation averaged 97 minutes (range: 10-138 min). The neurological evaluation of our patients included an assessment of stroke severity using the NIHSS, with a mean score of 9 (SD = 6.53). In all, 33.05% of our patients had a minor stroke, defined by a NIHSS <5 (CI: 28.81-37.56), while 22.46% had a severe stroke, with a NIHSS >21 (CI: 18.80-26.59). The mean ASPECT score was 8; 36.41% of the CT images were given a score of 10 (CI: 31.85-41.22), and 8.04% of the images were given a score ≤7 (CI: 6.31-14.05), which correlated to the established AIS.

## Discussion

Our eligibility rate for rTPA use remains very low. When compared with other studies, our rate exceeds ones reported by studies from other developing countries but remains lower than ones from developed countries, as summarised in Table 3. An onset-to-thrombolysis delay exceeding the recommended 4.5 hours remains the main exclusion criterium. Similar findings have been reported by many studies [16-20]. In several developing countries, this delay was reported to be 3 to 5 times longer than the recommended time frame. Late arrival to the hospital was found to be the main reason behind these numbers [21,22]. In our study, mean pre-hospital delay, defined as the delay between symptom-onset and admission to our centre, was 24.9 hours. Poor recognition of stroke symptoms by the general population and a lack of EMS use are considered the two main factors explaining this result. Patients who called the EMS after recognising stroke signs arrived to our centre with a much shorter delay than those who chose another means of transport. However, less than 15% of our patients used EMS. These findings emphasise the need for comprehensive public education in our city regarding stroke. Our efforts should promote the benefits of using the EMS and include a presentation of the services available in our region. Another factor explaining the late hospital arrival is previous patient admission in another healthcare facility without the infrastructure required for the management of stroke. It is hence of the utmost importance to dispatch stroke patients to the highest level of care available in the shortest time possible [23]. Consequently, our education efforts should stress the urgent need to transport patients to our facility when stroke is suspected. To improve the synergy between the pre-hospital and in-hospital phases, our procedures should also include advance notification of stroke patient arrival by EMS. This has been proven to shorten the time needed for the evaluation and time to brain imaging and thus led to increased use of thrombolysis [24].

**Table 3:** thrombolysis rates in different countries

Study	Period of the study	Thrombolysis rate (%)
**Suwanwela *et al*. (Thailand)**	**January-December 2006**	**2.1**
Ayromlou *et al*. (Iran)	January-December 2014	3.1
Man K *et al*. (Malaysia)	January-December 2006	4.8
Liu *et al*. (New Zealand)	January 2015-June 2016	7.0
**Our study**	January-August 2017	8.4
Sushma *et al*. (India)	January-December 2014	10.7
McCormick *et al*. (United Kingdom)	July 2004-July 2005	10.5
Sung *et al*. (Taiwan)	January 2010-September 2011	12.0
Dalloz *et al*. (France)	December 2005-July 2009	12.9
Scherf *et al*. (Netherlands)	January-December 2012	14.6
Reiff *et al*. (Switzerland)	January 2003-January 2011	22.9
Schwamm *et al*. (USA)	January 2003-January 2007	30.8

With regard to our in-hospital management, the mean door-to-CT evaluation delay was 138 minutes, exceeding the 60 minute time frame recommended by the AHA [13]. Similar results were reported by studies conducted in developing countries. Ayromlou *et al*. reported a delay of 91 minutes in Iran and Wasay *et al*. a delay of 120 minutes in Pakistan [16,19]. Our in-hospital management may also have been prone to delay since, in the end, no acute endovascular therapy was available. This highlights the need to devise several strategies to shorten our in-hospital delays. The gold standard in the management of stroke patients in hospital settings is known to be the ‘Helsinki Model’, with a mean in-hospital delay of 20 minutes [25]. Key components of the model, such as a ‘Code-Stroke’ pre-notification pathway, the transport of the patient on the EMS stretcher, priority access to brain imaging and thrombolysis initiation on the CT table [26], should be implemented in our services. An acute stroke team should also be made permanently available and simultaneously activated by the Code Stroke. All these interventions have proven to be transferrable in any emergency facility [27]. Another key aspect of our strategy should be the creation of a regional stroke register as part of a quality improvement process [13]. This would enable the identification of strengths and weaknesses of our system and allow the design of further implementation strategies for permanent optimisation of our work. It is important to mention that our study had two main limitations. First, the pre-hospital delays were recorded at patient admission to our facility, by asking the patient, an accompanying family member or the paramedics. This may have led to inaccuracies since some delays exceeded 24 hours from symptom-onset. Second, we did not consider the patients´ ability to afford the therapy. As many insurance companies in Morocco do not cover the expenses of thrombolysis, we believe that many patients would not benefit from it because of financial constraints, and, thus, our eligibility rates would certainly be lower.

## Conclusion

Most of our patients failed to meet the thrombolysis inclusion criteria because of an onset-to-thrombolysis delay longer than the recommended 4.5 hours, mainly due to pre-hospital delays far exceeding AHA/ASA guidelines. Public stroke education, optimisation of the EMS and in-hospital strategies are the core elements for shortening our delays and thus increasing our thrombolysis rate before implementing the therapy. For this matter, we need to set up a comprehensive stroke system of care in our region, integrating every actor in the stroke chain of survival, from the general public to the acute stroke team.

### What is known about this topic

- Thrombolysis with rTPA is an approved method for the treatment of acute ischaemic stroke;
- Due to its narrow therapeutic window, the use of rTPA remains low;
- Efforts are made to shorten pre-hospital and in-hospital delays in the management of patients with acute ischaemic stroke.

### What this study adds

- First study assessing eligibility for thrombolysis before implementing the therapy;
- Reasons for non-eligibility for thrombolysis in Morocco;
- Identification of strategies to be implemented to increase the rate for thrombolysis in a developing country such as Morocco.

## References

[1] Mukherjee D, Patil CG (2011). Epidemiology and the global burden of stroke. World Neurosurg.

[2] Feigin VL, Forouzanfar MH, Krishnamurthi R, Mensah GA, Connor M, Bennett DA (2014). Global and regional burden of stroke during 1990-2010 findings from the Global Burden of Disease Study 2010. Lancet.

[3] Lopez AD, Mathers CD, Ezzati M, Jamison DT, Murray CJL (2006). Global and regional burden of disease and risk factors 2001, systematic analysis of population health data. Lancet.

[4] Adeloye D (2014). An estimate of the incidence and prevalence of stroke in Africa: a systematic review and meta-analysis. PLoS ONE.

[5] Tran J, Mirzaei M, Anderson L, Leeder SR (2010). The epidemiology of stroke in the Middle East and North Africa. J Neurol Sci.

[6] Engels T, Baglione Q, Audibert M, Viallefont A, Mourji F, El Alaoui Faris M (2014). Socioeconomic status and stroke prevalence in Morocco: results from the Rabat-Casablanca study. PLoS ONE.

[7] Haršány M, Tsivgoulis G, Alexandrov AV (2014). Intravenous thrombolysis in acute ischemic stroke: standard and potential future applications. Expert Rev Neurother.

[8] Balami JS, Chen R, Sutherland BA, Buchan AM (2013). Thrombolytic agents for acute ischaemic stroke treatment: the past, present and future. CNS Neurol Disord Drug Targets.

[9] Wardlaw JM, Murray V, Berge E, del Zoppo GJ (2014). Thrombolysis for acute ischaemic stroke. Cochrane Database Syst Rev.

[10] Lees KR, Bluhmki E, von Kummer R, Brott TG, Toni D, Grotta JC (2010). Time to treatment with intravenous alteplase and outcome in stroke: an updated pooled analysis of ECASS, ATLANTIS, NINDS, and EPITHET trials. Lancet.

[11] de Los Ríos la Rosa F, Khoury J, Kissela BM, Flaherty ML, Alwell K, Moomaw CJ (2012). Eligibility for intravenous recombinant tissue-type plasminogen activator within a population: the effect of the European Cooperative Acute Stroke Study (ECASS) III Trial. Stroke.

[12] National Institute of Neurological Disorders and Stroke rt-PA Stroke Study Group (1995). Tissue plasminogen activator for acute ischemic stroke. N Engl J Med.

[13] Jauch EC, Saver JL, Adams HP, Bruno A, Connors JJB, Demaerschalk BM (2013). Guidelines for the early management of patients with acute ischemic stroke: a guideline for healthcare professionals from the American Heart Association/American Stroke Association. Stroke.

[14] Daouda MT, Bouchal S, Chtaou N, Midaoui A, Souirti Z, Belahsen F (2018). Thrombolysis alert in Hassan II University Teaching Hospital of Fez (Morocco): a prospective study of 2 years. Journal of Stroke and Cerebrovascular Diseases.

[15] Kharbach A, Obtel M, Lahlou L, Aasfara J, Mekaoui N, Razine R (2019). Ischemic stroke in Morocco: a systematic review. BMC Neurol.

[16] Wasay M, Barohi H, Malik A, Yousuf A, Awan S, Kamal AK (2010). Utilization and outcome of thrombolytic therapy for acute stroke in Pakistan. Neurol Sci.

[17] Scherf S, Limburg M, Wimmers R, Middelkoop I, Lingsma H (2016). Increase in national intravenous thrombolysis rates for ischaemic stroke between 2005 and 2012 is bigger better?. BMC Neurol.

[18] Balami JS, Hadley G, Sutherland BA, Karbalai H, Buchan AM (2013). The exact science of stroke thrombolysis and the quiet art of patient selection. Brain.

[19] Ayromlou H, Soleimanpour H, Farhoudi M, Taheraghdam A, Sadeghi Hokmabadi E, Rajaei Ghafouri R (2014). Eligibility assessment for intravenous thrombolytic therapy in acute ischemic stroke patients; evaluating barriers for implementation. Iran Red Crescent Med J.

[20] Eissa A, Krass I, Levi C, Sturm J, Ibrahim R, Bajorek B (2013). Understanding the reasons behind the low utilisation of thrombolysis in stroke. Australas Med J.

[21] Walker RW, Rolfe M, Kelly PJ, George MO, James OFW (2003). Mortality and recovery after stroke in the Gambia. Stroke.

[22] Brainin M, Teuschl Y, Kalra L Acute treatment and long-term management of stroke in developing countries. The Lancet Neurology. 2007 Jun 1;.

[23] Mohammad YM (2008). Mode of arrival to the emergency department of stroke patients in the United States. J Vasc Interv Neurol.

[24] Abdullah AR, Smith EE, Biddinger PD, Kalenderian D, Schwamm LH (2008). Advance hospital notification by EMS in acute stroke is associated with shorter door-to-computed tomography time and increased likelihood of administration of tissue-plasminogen activator. Prehosp Emerg Care.

[25] Meretoja A, Strbian D, Mustanoja S, Tatlisumak T, Lindsberg PJ, Kaste M (2012). Reducing in-hospital delay to 20 minutes in stroke thrombolysis. Neurology.

[26] Powers WJ, Rabinstein AA, Ackerson T, Adeoye OM, Bambakidis NC, Becker K (2018). 2018 Guidelines for the Early Management of Patients with Acute Ischemic Stroke: A Guideline for Healthcare Professionals from the American Heart Association/American Stroke Association. Stroke.

[27] Wu TY, Coleman E, Wright SL, Mason DF, Reimers J, Duncan R (2018). Helsinki Stroke Model is transferrable with “real-world” resources and reduced stroke thrombolysis delay to 34 min in Christchurch. Front Neurol.

